# Chronology of cellular events related to mitochondrial burnout leading to cell death in Fuchs endothelial corneal dystrophy

**DOI:** 10.1038/s41598-020-62602-x

**Published:** 2020-04-02

**Authors:** Sébastien J. Méthot, Stéphanie Proulx, Isabelle Brunette, Patrick J. Rochette

**Affiliations:** 1 Centre de Recherche du CHU de Québec – Université Laval, Axe Médecine Régénératrice, Hôpital du Saint-Sacrement, Québec, Canada; 2 Centre de recherche en organogénèse expérimentale de l’Université Laval/LOEX, Québec, Canada; 3 Université Laval, Faculté de Médecine, Département d’Ophtalmologie, Université Laval, Québec, Canada; 4 Maisonneuve-Rosemont Hospital Research Center, Montreal, Québec, Canada; 5 Department of Ophthalmology, University of Montreal, Montreal, Québec, Canada

**Keywords:** Mechanisms of disease, Mitochondria

## Abstract

Fuchs endothelial corneal dystrophy (FECD) is a degenerative eye disease characterized by corneal endothelial cell (CEC) death and the formation of guttae, an abnormal thickening of CEC’s basement membrane. At the tissue level, an oxidative stress causing mitochondrial damage and CEC death have been described to explain FECD pathogenesis. At the cellular level, our group has previously observed significant variability in the mitochondrial mass of FECD CECs. This led us to hypothesize that mitochondrial mass variability might play a key role in the chronology of events eventually leading to CEC death in FECD. We thus used different fluorescent markers to assess mitochondrial health and functionality as a function of mitochondrial mass in FECD corneal endothelial explants, namely, intra-mitochondrial calcium, mitochondrial membrane potential, oxidation level and apoptosis. This has led us to describe for the first time a sequence of events leading to what we referred to as a mitochondrial burnout, and which goes as follow. FECD CECs initially **compensate** for endothelial cell losses by incorporating mitochondrial calcium to help generating more ATP, but this leads to increased oxidation. CECs then **resist** the sustained need for more ATP by increasing their mitochondrial mass, mitochondrial calcium and mitochondrial membrane potential. At this stage, CECs reach their maximum capacity and start to cope with irreversible oxidative damage, which leads to mitochondrial **burnout**. This burnout is accompanied by a dissipation of the membrane potential and a release of mitochondrial calcium, which in turn leads to cell **death** by apoptosis.

## Introduction

The cornea is composed of three cellular layers, i.e. the epithelium, the stroma and the endothelium^1^. The stroma needs to be maintained partially dehydrated to allow for the optimal arrangement of its collagen fibrils necessary to ensure its transparency^2^. This ATP-demanding function is achieved mainly by the corneal endothelium, a leaky cell monolayer that pumps fluid out of the stroma, into the anterior chamber, maintaining the proper hydration of the corneal stroma^2,3^. Fuchs endothelial corneal dystrophy (FECD) is characterized by a loss of corneal endothelial cells (CECs), abnormal extracellular matrix deposition on its basal membrane (Descemet’s membrane) leading to the formation of excrescences called *guttae*, and changes in cellular morphology^4^. The loss of CECs ultimately compromises the role of the endothelium to preserve corneal deturgescence. This leads to corneal edema and vision loss^4^. FECD affects 4 percent of the US populations over 40 years old^5^ and the only treatment currently available for FECD is corneal transplantation^6,7^.

Although the exact cause of FECD is not yet determined, there is growing evidence that oxidative stress and mitochondria are major contributors^8,9^. The mechanism and the sequence of events leading to mitochondrial dysfunction and cell loss still remain to be elucidated. Manifestations of cumulative oxidative damage and mitochondrial dysfunction in FECD cells have been described by our group and others, including mtDNA deletion accumulation and telomere shortening^9–11^ and it is known that when mitochondrial damage is severe enough to cause a loss of function, apoptosis is triggered^12^. Indeed, using an immortalized cultured endothelial cell line as model of FECD, it has been previously shown that increasing endogenous oxidative stress via menadione treatment leads to rosette formation, mitochondrial dysfunction and activation of apoptosis in FECD cells^13^. Moreover, subsequent work by the same group showed that the loss of mitochondria in FECD would be caused by the activation of mitophagy^14^. We also know that since CECs do not proliferate *in vivo*, the remaining cells adapt their morphology to occupy the space left by the dead cells in the tissue^8,15^. Moreover, many genes coding for mitochondrial antioxidant have been found downregulated in FECD^8^. Thus, in addition to being more exposed to oxidative stress via the decompensation of CECs, the mitochondrial antioxidant defenses of FECD cells are also less effective to cope with this oxidative stress.

**Figure 1:**
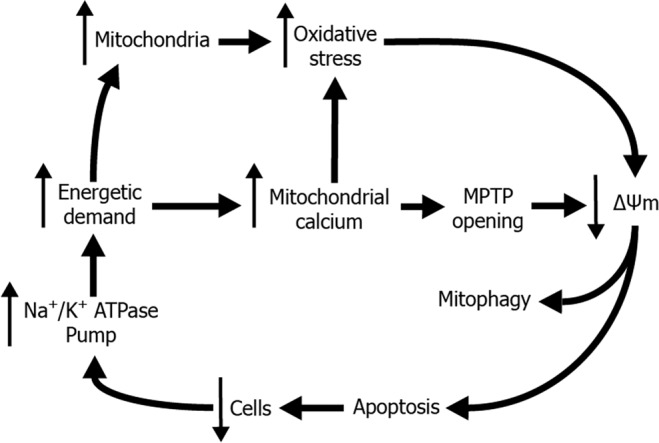
Vicious cycle of FECD pathogenesis. The CEC Na+K+−ATPase pump, required to maintain corneal deturgescence, uses 30% of the cellular ATP, this high energetic demand is met by increasing the number of mitochondria and/or by increasing mitochondrial calcium intake. However, this leads to oxidative stress and/or the formation of mitochondria permeability transition pores [MPTP], resulting in a loss of mitochondrial membrane potential [↓Δψm] and elimination of altered mitochondria by mitophagy or induction of cell death by apoptosis. When FECD CEC die [↓ Cells], the amount of energy globally required to maintain corneal deturgescence remains the same, therefore increasing ATP demand on remaining cells, in turn promoting mitochondrial exhaustion. Although all cells are not affected to the same degree, each additional cell death feeds the vicious cycle.

Based on what is known in the literature, we have derived a hypothetical vicious circle in which the mitochondrion is central. (Fig. 1). Mitochondrial oxidative stress and/or the reduced capacity to cope with oxidation lead to a loss of mitochondrial membrane potential (ΔΨm)^16^. The loss of ΔΨm leads to an increase mitochondrial recycling (mitophagy) and apoptosis^16,17^. When FECD CECs die, the amount of ions globally required to maintain stromal deturgescence remains the same, thus increasing the energetic demand and Na^+^/K^+^ ATPase pump on each remaining cell^18^. The resulting increase in ATP demand at the cellular level leads to an increase in mitochondrial mass and mitochondrial calcium. Mitochondrial mass and calcium increase the amount of ATP available by respectively making more components of the electron transport chain and making these components more efficient^19–21^. As a consequence, oxidative phosphorylation is increased, as well as the production of by-products such as reactive oxygen species (ROS), resulting in oxidative stress^22–24^. Elevated mitochondrial calcium and oxidative stress also result in the opening of mitochondrial permeability transition pores, which dissipates the ΔΨm^25,26^.

In this hypothetic vicious circle, mitochondria allow for a compensation mechanism for cell depletion, but this eventually leads to an energetic exhaustion that we call “mitochondrial burnout”. We previously showed that, in the same FECD explant, individual CECs present different levels of mitochondrial mass^10^, suggesting that each CEC is not at the same stage of the vicious circle. In an attempt to decipher the chronology of events that leads to cell death in FECD, we analyzed the different elements of the vicious circle using human FECD corneal endothelial explants. We used mitochondrial mass as a marker of progression of individual CECs in the vicious circle and we assessed different cellular markers of mitochondrial and cellular functionality, i.e. oxidation level, apoptosis, ΔΨm and mitochondrial calcium. The great variation in mitochondrial mass within the same FECD explant was confirmed. A lower mitochondrial mass was associated with apoptotic CECs and a loss of ΔΨm, while a higher mass was associated with oxidative stress. An elevated level of mitochondrial calcium was also found in FECD explants. Taken together, our results allow to describe a sequence of events leading to mitochondrial exhaustion and ultimately to the death of FECD CECs.

## Materials and Methods

All experiments in this study were performed in accordance with the Declaration of Helsinki, and the research protocol received approval by the CIUSSS-EMTL (Montréal) and the CHU de Québec-Université Laval (Québec) institutional ethics committees for the protection of human subjects with written informed patient consent for study participation. No tissue samples were procured from prisoners. FECD explants (corneal endothelium and Descemet’s membrane) were collected from 13 consenting patients (age 55–90 years; median 70; SD ± 9.3) with late stage FECD at the time of their corneal transplantation (Centre universitaire d’ophtalmologie (CUO), Hôpital Maisonneuve-Rosemont (HMR) and CUO – Hôpital du Saint-Sacrement, Québec, Canada). Twelve healthy corneas (age 56–82 years; median 67; SD ± 9.8) unsuitable for transplantation were obtained from our local eye bank (Banque d’Yeux du Centre universitaire d’ophtalmologie, Québec, Canada) and their Descemet’s membrane (with attached endothelium) were isolated. Every cellular portion of each FECD explant was analyzed and no discrimination between central and peripheral portion of the explant was done. Age, sex and clinical diagnosis of all donors from whom corneal tissue was used in this study are listed in Supplementary Material (Table S1).

### Markers used on FECD and healthy endothelium explants

Corneal endothelium on their Descemet’s membrane were kept overnight at 37 °C in growth medium, as previously described^10^. Explants were then washed with Opti-Mem-I (for all markers but CM-H_2_DCFDA; Invitrogen, Burlington, ON, Canada) or PBS (for CM-H_2_DCFDA). Mitotracker Deep Red FM (Invitrogen) was used in combination with JC-1 (Invitrogen), CellEvent Caspase-3/7 Green Detection Reagent (Invitrogen) and CM-H_2_DCFDA (Invitrogen) at a concentration of 80 nM. Mitotracker Green FM (Invitrogen) was used with Rhod-2 AM at a concentration of 80 nM. JC-1 was used to measure mitochondrial membrane potential at a concentration of 2.5 μM. Rhod-2 AM was used to measure mitochondrial calcium, at a concentration of 3 μM. CellEvent Caspase-3/7 Green Detection was used to measure caspase-3/7 activity marker, at a concentration of 5 μM. CM-H_2_DCFDA was used to measure ROS presence, at a concentration of 2.5 μM. The explants were put in staining solution of PBS (for CM-H_2_DCFDA), PBS with 5% fetal bovine serum (for caspase-3/7 activity marker) or Opti-Mem-I (for JC-1 and Rhod-2) containing the markers with their appropriate mitochondrial probe. The explants were then incubated for 30 minutes at 37 °C and 8% CO_2_, washed with Opti-Mem-I or PBS, put on a microscope slide, observed and imaged. Solvents for each marker (PBS, PBS with 5% FBS or Opti-Mem-1) was determined according to the manufacturer’s recommendations.

### Image analysis

The signal produced by the different markers was quantified using AxioVision 4.8.2 software (Zeiss, Germany). Cells were individually analyzed for each marker and their corresponding mitochondrial staining. Background was measured and removed from the signal of the different markers.

### Statistical analysis

To generate statistical analysis, Kaleida graph version 4.1.3 software (Synergy software, Readint, PA, USA) was used. Differences between groups were assessed with the two-tailed homoscedastic Student’s t-test. A p-value ≤ 0.05 was defined as statistically significant. Differences in variation between groups were assessed by comparing the coefficient of variation (the ratio between the standard deviation and the mean). Box plot showing median values has been used to present data.

## Results

### Mitochondrial mass variability is increased in FECD CECs compared to Healthy CECs

**Figure 2:**
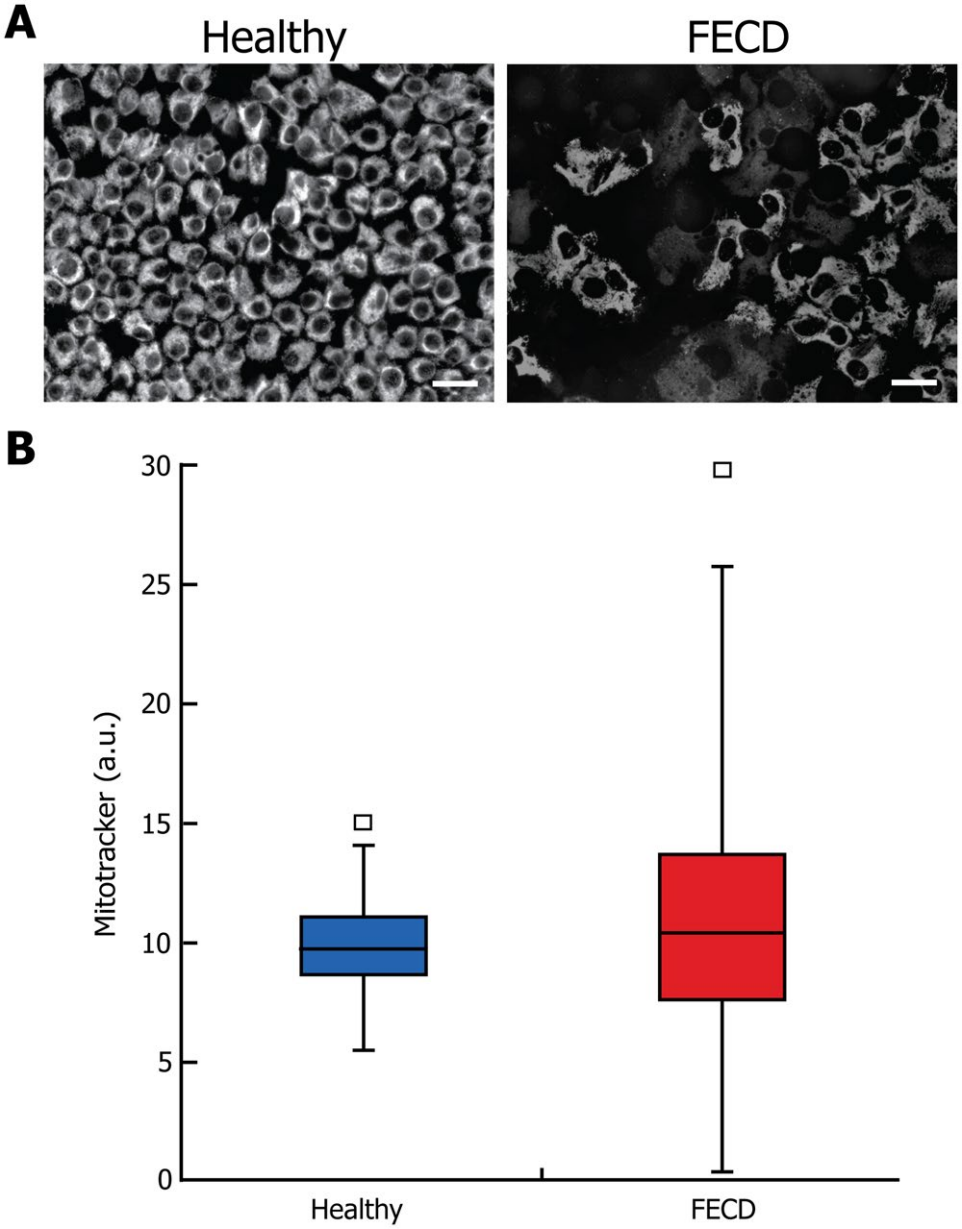
Higher variability of mitochondrial mass in FECD explants. (**A**) A marker of mitochondrial mass (mitotracker) was used in healthy and FECD endothelial explants. The typical CEC loss and the enlargement of remaining CECs in the FECD explants (right) compared to the healthy explants (left) is well illustrated. (**B**) Quantification of the mitotracker signal showed no statistically significant difference in mitochondrial mass between healthy and FECD explants. However, FECD cells presented more variation (Coefficient of variation (CV) = 0.44) than the Healthy cells (CV = 0.17). Scale bar = 40 μm. Median and standard deviation are reported. Experiments were performed with 3 different explants from Healthy (1200 cells analyzed, N = 3) and 3 FECD patients (1176 cells analyzed, N = 3).

The mitochondrial mass was measured FECD explants and compared it to healthy endothelial explants. An important variability in mitochondrial mass was observed among CECs of each FECD explant (Fig. 2A). This was corroborated by the quantification of the Mitotracker signal (Fig. 2B). Median mitochondrial mass of healthy (9.7 a.u.) and FECD (10.4 a.u.) explants were not statistically significantly different, but FECD CECs presented more variation (Coefficient of variation (CV) = 0.44) than Healthy CEC (CV = 0.17).

### Caspase-3/7 activity is linked to mitochondrial mass depletion in FECD CECs

**Figure 3:**
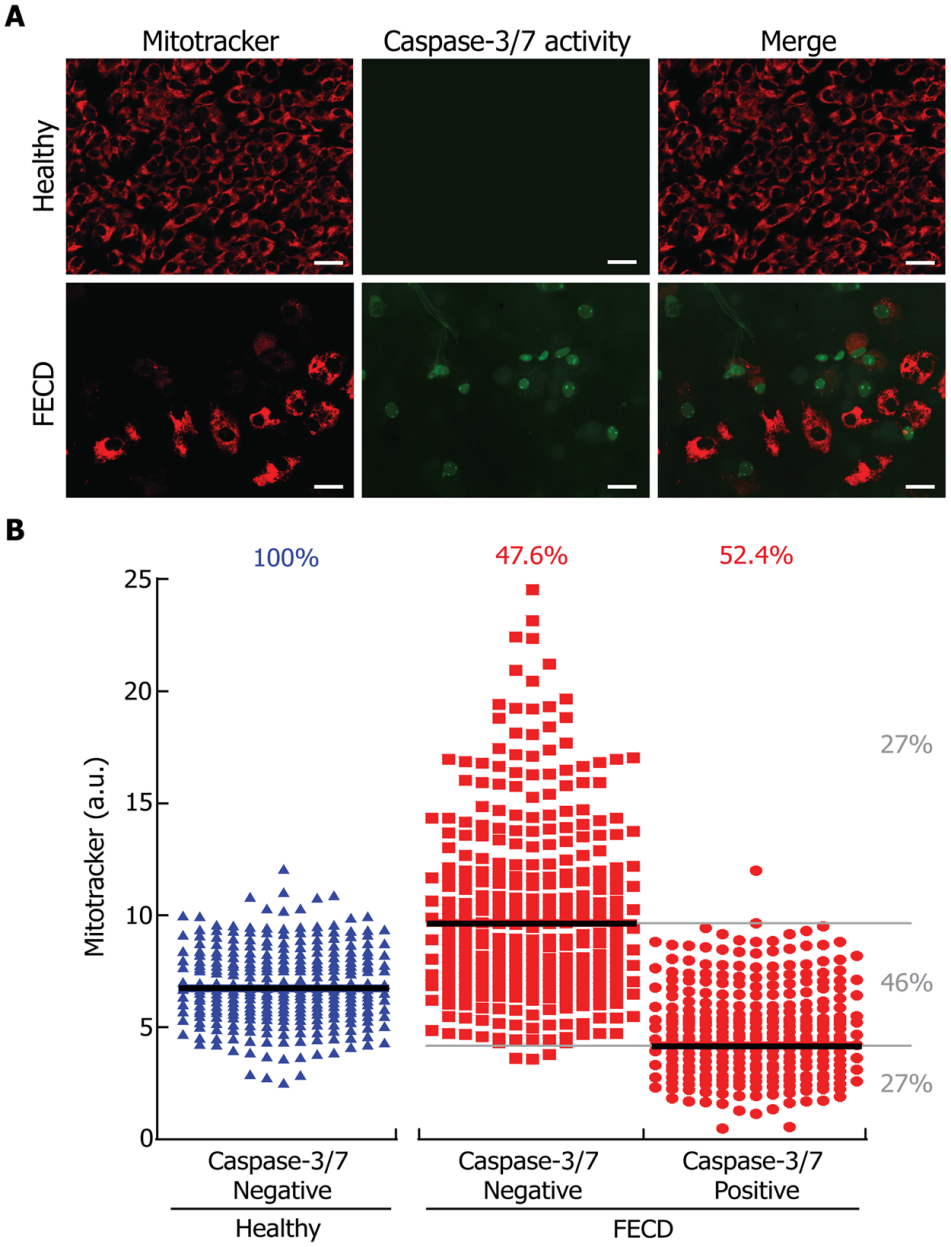
Cell death is linked to mitochondrial mass depletion in FECD CEC. (**A**) A marker of cell death by apoptosis (caspase-3/7 activity; green) was used in conjunction with the mitochondrial mass marker (mitotracker; red) in healthy and FECD explants. (**B**) Mitochondrial and caspase-3/7 signals were quantified in healthy (blue) and FECD (red) explants and plotted. Each dot represents a single cell. No caspase-3/7 signal was detected in healthy explants. Caspase-3/7 negative and positive cells composed 48% and 52% of the FECD cells, respectively. The median mitochondrial mass was higher in caspase-3/7 negative cells than in caspase-3 positive cells. FECD CECs with mitochondrial mass ranging between Caspase-3/7 positive and negative medians composed 46% of the FECD cellular population. These cells are referred to as containing a “Normal mitochondrial level”, which happened to be very similar to that of healthy cells. Twenty seven percent of the Caspase-3/7 negative FECD cells were above the median mitochondrial mass level and are therefore referred to as containing a “High mitochondrial level”. On the other hand, 27% of the caspase-3/7 positive FECD cells were below the median mitochondrial mass level and are therefore referred to as containing a “Low mitochondrial level”. Scale bar = 40 μm. Experiments were performed with 3 different explants from Healthy (900 cells analyzed, N = 3) and 3 FECD patients (846 cells analyzed, N = 3).

We measured caspase-3/7 activity as a direct marker of apoptosis in conjunction with mitochondrial mass to determine if there is a correlation between mitochondria depletion and cell death in FECD (Fig. 3A). A positive control of caspase 3/7 apoptosis labeling can be found in Supplementary Material (Fig. S1). The mitochondrial mass signal measured for caspase-3/7 positive and negative cells is presented in Fig. 3B. All cells in healthy explants were caspase-3/7 negative, while in the FECD explants, only 48% of the cells were caspase-3/7 negative and 52% were caspase-3/7 positive. The median mitochondrial mass was higher (9.6 a.u.) in caspase-3/7 negative than in caspase-3/7 positive (4.1 a.u.) FECD cells. Mitochondrial mass (6.7 a.u.) in healthy cells was intermediate between that of caspase-3/7 positive and caspase-3/7 negative FECD CECs. A small percentage (9%) of FECD cells were caspase-3/7 positive without a detectable mitochondrial mass. Cells with mitochondrial mass between caspase-3/7 positive and negative medians represented 46% of FECD CECs and were referred to as having a “normal mitochondrial mass”. Caspase-3/7 negative cells with a mitochondrial mass above the “normal level” composed 27% of the FECD CECs and were therefore referred to as “High mitochondrial mass”. Caspase-3/7 positive FECD cells with a mitochondrial mass below the “normal level” composed 27% of the cells therefore referred to as “Low mitochondrial mass”. These results indicate that an association can be made between mitochondrial mass and cell death in FECD CECs, as cells with lower mitochondrial mass were apoptotic, while cells with higher mitochondrial mass were not apoptotic.

### Oxidative stress correlates with mitochondrial mass level in FECD CECs

**Figure 4:**
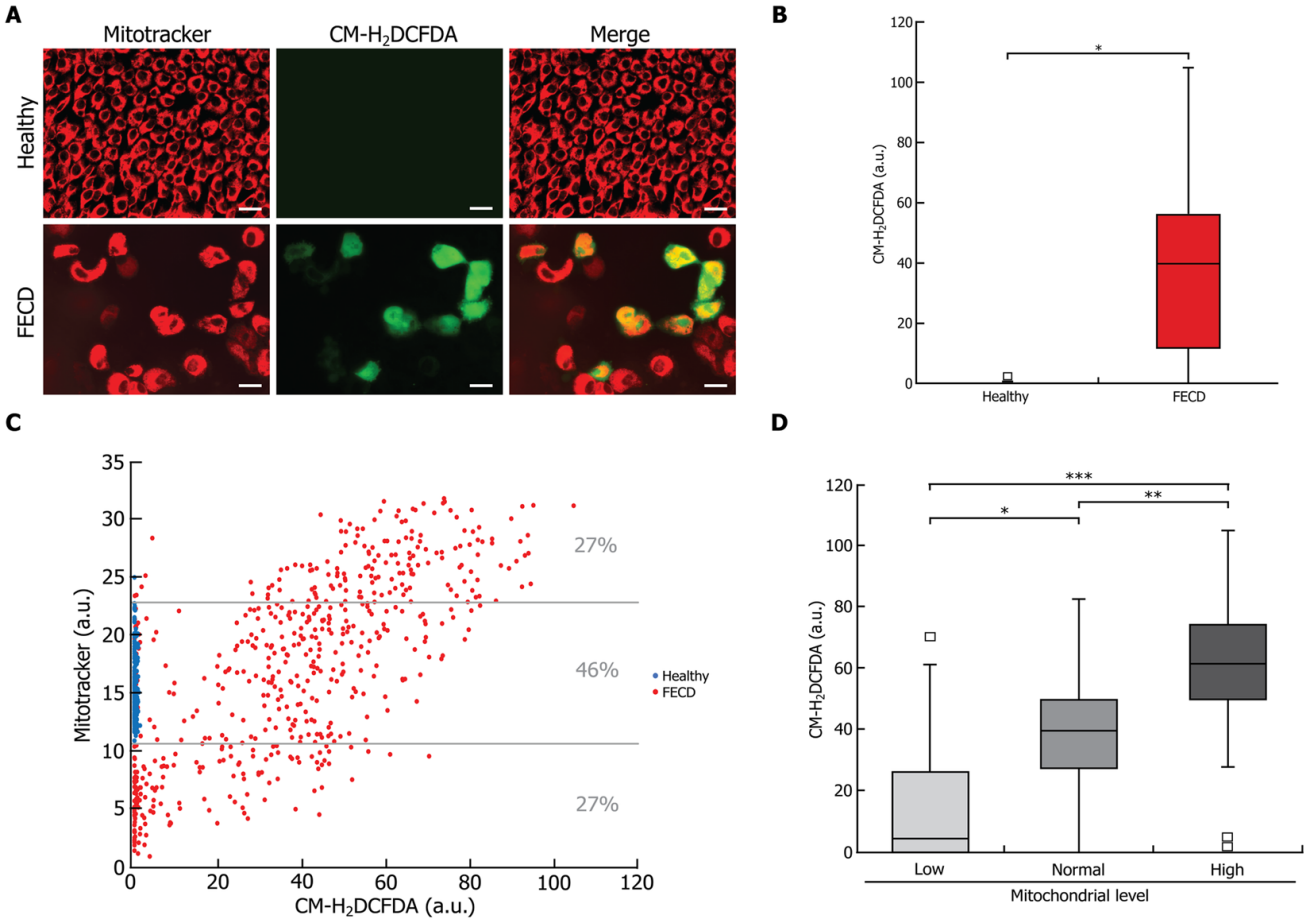
Oxidative stress correlates with an increase in mitochondrial mass in FECD cells. (**A**) A marker of oxidative stress (CM-H_2_DCFDA; green) was measured in conjunction with mitochondrial mass marker (mitotracker; red) in healthy and FECD. (**B**) Quantification of the oxidative stress marker showed no detectable level of oxidation in healthy endothelial explants, but a significantly higher amounts of oxidation in CECs of FECD explants. (**C**) CM-H_2_DCFDA and mitotracker signal levels were plotted and each dot represents a single CEC (blue for healthy cells and red for FECD). A strong positive correlation was found between the mitochondrial mass and the oxidative stress level (R^2^ = 0.514; p < 0.0001). (**D**) Quantification of CM-H_2_DCFDA in FECD CEC populations according to their mitotracker level, *i.e*. low, normal and high mitochondrial level categories as depicted in Fig. 3. The significant difference in oxidation level between low and normal, as well as between normal and high categories confirms that a higher level of mitochondrial mass is associated with a higher level of oxidative stress, and vice versa. Scale bar = 40 μm. Experiments were performed with 3 different explants from Healthy (600 cells analyzed, N = 3) and 3 FECD patients (594 cells analyzed, N = 3). *p < 0.01;**p < 0.001;***p < 0.0001.

We measured the oxidative stress in conjunction with the mitochondrial mass in order to determine whether an association can be made between mitochondrial mass and ROS accumulation. CM-H_2_DCFDA labeling of ROS clearly showed an oxidative stress in FECD CECs, while none was detectable in healthy CECs (Fig. 4A). Signal quantification of CM-H_2_DCFDA also demonstrated that the ROS level was statistically significantly higher in FECD (36.8 a.u.) than in healthy (0.6 a.u.) cells (Fig. 4B). However, 24.5% of the FECD cells presented very low ROS levels, comparable to those found in healthy cells.

A strong positive correlation was observed between oxidative stress level and mitochondrial mass (R^2^ = 0.514; p < 0.0001) (Fig. 4C). We then categorized FECD cells according to their mitochondrial mass following the same 3 categories described above for caspase-3/7 activity (*i.e*. High (27% of FECD cells), Normal (46%) and Low (27%) mitochondrial mass). As expected, the level of mitochondria in healthy cells fell into the “Normal mitochondrial mass” category. Median levels of CM-H_2_DCFDA in each category were: High: 61.3 a.u., Normal: 39.5 a.u., Low: 4.4 a.u. (Fig. 4D). This confirmed that a higher level of mitochondrial mass is associated with a higher level of oxidative stress. Moreover, since mitochondrial levels strongly correlate with ROS levels, it can be extrapolated from the associations documented between mitochondrial mass and caspase-3/7 (Fig. 3) that FECD cells with lower ROS levels are apoptotic while those with higher ROS levels are not apoptotic.

### Mitochondrial calcium levels are increased in FECD CECs compared to healthy CECs

**Figure 5:**
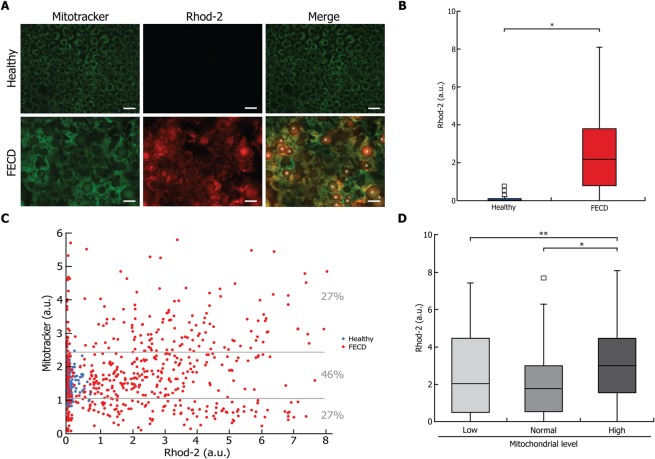
Higher mitochondrial calcium is found in FECD cells. (**A**) A marker of intra-mitochondrial calcium (Rhod-2; red) was measured in conjunction with mitochondrial mass marker (mitotracker; green) in healthy and FECD explants. Rhod-2 labelling allows the observation that CECs were much larger in the FECD than in the healthy explant and numerous guttae (star) are seen, disorganizing the architecture of the endothelial mosaic. (**B**) Quantification of intra-mitochondrial calcium showed a significantly higher levels of calcium in the FECD CECs, with no detectable level in healthy CECs. (**C**) Rhod-2 and mitotracker signal levels were plotted, each dot representing a single CEC (blue for healthy cells and red for FECD). A weak correlation was observed (R^2^ = 0.020; p < 0.001). (**D**) Quantification of Rhod-2 in FECD CEC populations according to their mitotracker mass, i.e. low, normal and high mitochondrial level categories as depicted in Fig. 3, revealed that only the category with a high mitochondrial mass had a calcium level significantly higher than that of the other two categories. Scale bar = 40 μm. Experiments were performed with 3 different explants from Healthy (800 cells analyzed, N = 3) and 4 FECD patients (710 cells analyzed, N = 4). *p < 0.01 and **p < 0.001.

Mitochondrial calcium was then measured in conjunction with mitochondrial mass in order to determine whether there was an association between the two. Rhod-2 labeling of intra-mitochondrial calcium was higher in FECD than in Healthy CECs (Fig. 5A). This was confirmed by signal quantification, with mitochondrial calcium levels significantly higher in FECD (median: 2.2 a.u.) than in healthy (0 a.u.) cells (Fig. 5B). However, 24.2% of FECD CECs presented a mitochondrial calcium level comparable to that of the healthy cells. The mitochondrial calcium level was plotted against mitochondrial mass (Fig. 5C), and a weak correlation was observed (R^2^ = 0.020; p < 0.001). FECD cells were then categorized according to their mitochondrial level following the same 3 categories determined for caspase-3/7 activity (Fig. 5D). This revealed that only the category with High mitochondrial mass had a significantly higher level of calcium than the other 2 categories. We may extrapolate from the associations documented between mitochondrial mass and caspase-3/7 (Fig. 3), that a high intra-mitochondrial calcium is associated with FECD cell death.

### Dissipation of ΔΨm in FECD CEC and its correlation with mitochondrial mass

**Figure 6:**
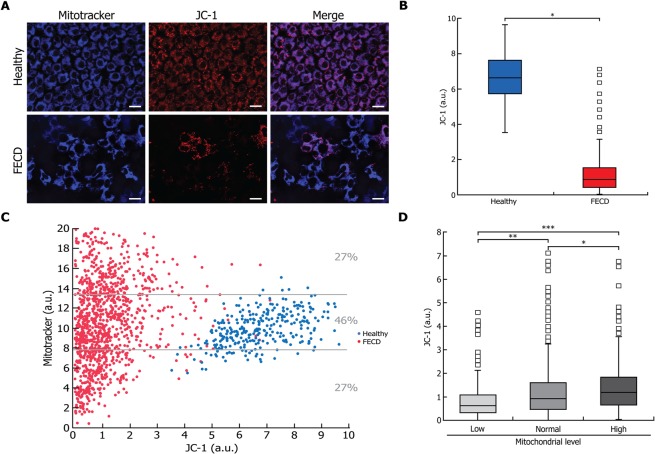
Mitochondrial membrane potential (ΔΨm) is lower in FECD and correlates with an increase in mitochondrial mass. (**A**) A marker of ΔΨm (JC-1; red) was measured in conjunction with mitochondrial mass marker (mitotracker; blue) in FECD and in healthy explants as controls. (**B**) Quantification of ΔΨm showed a high level of membrane potential in healthy cells, indicating of a normal capacity to generate ATP. On the other hand, a clear qualitative decrease in ΔΨm in FECD CEC, indicating a lower capacity to produce ATP in these cells. (**C**) Signal of ΔΨm and mitotracker mass were plotted, each dot representing a single CEC (blue for healthy cells and red for FECD). A weak positive correlation was found between mitochondrial mass and ΔΨm in healthy cells (R^2^ = 0.235; P < 0.0001) while that correlation was negligible in FECD cells (R^2^ = 0.038; p < 0.0001). (**D**) Distribution of the ΔΨm values of FECD CECs according to their mitotracker mass category (low, normal and high) as depicted in Fig. 3, revealed that ΔΨm was lowest in the “Low” and highest in the “High” mitochondrial level cell populations. Scale bar = 40 μm. Experiments were performed with 3 different explants from Healthy (1176 cells analyzed, N = 3) and 3 FECD patients (1200 cells analyzed, N = 3). *p < 0.01; **p < 0.001;***p < 0.0001.

ΔΨm was measured in conjunction with mitochondrial mass in order to determine whether there was an association between mitochondrial mass and ΔΨm. JC-1 staining of the ΔΨm showed a high variability among FECD CECs, unlike healthy cells (Fig. 6A). Quantification of the ΔΨm signal showed a median ΔΨm significantly lower in FECD (0.8 a.u.) than in healthy (6.6 a.u.) cells (Fig. 6B). A small portion (4%) of the FECD CECs, however, retained a ΔΨm level comparable to that of healthy CECs.

ΔΨm was then plotted against mitochondrial mass (Fig. 6C) and although statistically significant in both groups, the positive correlation observed between mitochondrial mass and ΔΨm was only mild for healthy CECs (R^2^ = 0.235; P < 0.0001) and negligible for FECD CECs (R^2^ = 0.038; p < 0.0001). When FECD CECs were categorized according to their mitochondrial mass as for the capase-3/7 activity, ΔΨm was found to be lowest in the “Low” and highest in the “High” mitochondrial mass cell populations (Fig. 6D). It can thus be extrapolated from the associations documented between mitochondrial mass and caspase-3/7 (Fig. 3) that cells with a lower ΔΨm are apoptotic and the one with high ΔΨm level are non-apoptotic. This means that a loss in ΔΨm would be associated with cell death in FECD cells.

## Discussion

In this study, we provide evidence about the chronology of a series of cellular events leading to mitochondria exhaustion and CEC death in FECD. The mitochondrial mass variability in FECD CECs (Fig. 2) strongly suggest that each individual cell is at different stage of the pathology. We used this variability in mitochondrial content to help us draw a clearer picture of the evolution of the pathology.

Our hypothesis, which is a more detailed version of the preliminary vicious cycle previously described^8,27^, is depicted in Fig. 1. FECD CEC loss by cell death induces an increase in the level of mitochondria in the remaining living CECs. Each remaining cell increases its mitochondrial mass in order to fill the increased ATP demand necessary to preserve proper deturgescence through the ATP-dependent ion pumping process^28,29^. But sustaining a high level of ATP production puts a strain on the cells and results in mitochondrial exhaustion/burnout^22–24^. There eventually is a depletion in mitochondria via mitophagy that remove non-functional mitochondria^17^.

In order to demonstrated this hypothesis, we categorized FECD CECs in 3 categories according to their mitochondrial content, i.e. Low, Normal and High (Fig. 3). Using these categories, we determined the level of different markers of mitochondrial and cellular health in an attempt to decipher the series of event occurring in FECD pathology.

We showed that FECD CECs containing a high mitochondrial mass are not apoptotic (Fig. 3), that they contain higher amounts of mitochondrial calcium (Fig. 5), higher amounts of ROS (Fig. 4) and that their ΔΨm is lower than healthy CECs (Fig. 6). These FECD cells are “resisting”, by increasing their ATP production in response to the loss of surrounding cells. The higher mitochondrial calcium could be a consequence of a high energetic need, which drives calcium uptakes, but without mitochondrial permeability transition pore opening to drive the exit^20,26^. This high mitochondrial mass and mitochondrial calcium lead to an increased in ROS production through oxidative phosphorylation by-products^22–24^. The lower ΔΨm is most likely a consequence of the sustained increase in ROS causing mitochondrial damage and affecting their function^30^. These FECD CECs with high mitochondrial mass are thus “resisting” but their near future is compromised by the sustained production of ROS and the increase in intra-mitochondrial calcium. Mitochondria will eventually reach the burnout state, which will lead to cell death.

We also showed that FECD CECs containing a low mitochondrial mass are apoptotic (Fig. 3), they show an increase in mitochondrial calcium (Fig. 5), a lower ROS content (Fig. 4) and the lowest ΔΨm of all categories (Fig. 6). Mitochondria have reached the burnout state and the CECs are dying. Very low ΔΨm indicates that their mitochondrial functionality is affected because of sustained ROS damaging and apoptotic signal^12,30^. These mitochondria are most likely eliminated by mitophagy, which explains their low amount. This is in agreement with previously published work showing that an increase in endogenous oxidative stress in endothelial cells leads to mitochondrial damage, loss in ΔΨm, activation of apoptosis and mitophagy^14^. The apoptotic cells might also be losing their ΔΨm because of an opening of mitochondrial permeability transition pore^31^. The low ROS level is the result of lower mitochondrial mass reducing the production of oxidative phosphorylation by-products^22–24^. While the mitochondrial calcium level is higher than Healthy cells, it is lower than the “High mitochondrial mass” category most likely because of an opening of the mitochondrial permeability transition pore^20,26^.

FECD CECs containing normal mitochondria mass showed characteristics between those of cells with low and high mitochondrial mass: (i) A similar proportion of apoptotic and non-apoptotic cells was observed in this category (Fig. 3); (ii) the levels of ROS and ΔΨm were higher than for the “low mitochondrial mass” but lower than the “high mitochondrial mass” categories of CECs (Figs. 4 and 6); and (iii) they show a decrease in mitochondrial calcium compared to the “high mitochondrial” containing CEC but similar to the “low”. FECD CECs containing a relative normal level of mitochondria are in fact transitioning from the “high mitochondrial” containing CEC to the “low” or from unaffected to the “high”.

**Figure 7:**
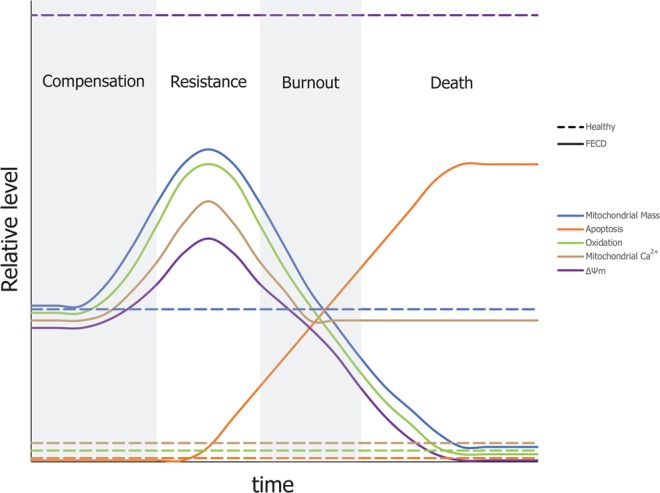
Theory of mitochondrial burnout leading to CEC death in FECD. Theoretical series of events related to the burnout stages leading to FECD CEC cell death. The relative level of each markers investigated in this study is plotted against a timeline. (**1**) **Compensation stage**: Normal mitochondrial mass, high mitochondrial calcium and high oxidative stress. No sign of apoptosis. FECD CEC in this stage are compensating for the loss of surrounding cells by incorporating mitochondrial calcium to help providing more ATP. The consequence is an increase in oxidative stress which leads to a lower ΔΨm. (**2**) **Resistance stage**: Further increase in mitochondrial mass, mitochondrial calcium, oxidative stress and membrane potential. FECD CEC in this stage are resisting the sustained need for more ATP caused by depletion of surrounding cells but they have reached their maximum capacity. The increase in mitochondrial mass leads to an increase in oxidative stress. (**3**) **Burnout stage**: Apoptotic cells with normal mitochondrial mass, lower ΔΨm, oxidation, and mitochondrial calcium. Mitochondrial permeability transition pore are most likely opening, which leads to a loss in ΔΨm and mitochondrial calcium. This leads to apoptosis of FECD CEC. (**4**) **Death stage**: Apoptotic CEC FECD with low mitochondrial mass, low oxidation stress and depleted ΔΨm. Alteration to mitochondria leads to a loss in mitochondrial mass through mitophagy and their loss of ΔΨm through the opening of the mitochondrial permeability transition pore.

Using the observations we made on FECD endothelial cell and mitochondria functional status, we were able to describe different states of FECD CEC functionality, from fully functionalilty to dead. We then categorized FECD CECs using the investigated markers according to the burnout stages (Fig. 7). (**1**) **Compensation stage**: FECD CECs containing a normal mitochondrial mass, high mitochondrial calcium and high oxidative stress but that have not shown apoptotic sign. These cells are compensating for the loss of cell in FECD by incorporating mitochondrial calcium to help providing more ATP. This results in an oxidative stress, which leads to a decrease in ΔΨm. (**2**) **Resistance stage**: FECD CECs containing the highest mitochondrial mass, a further increased in mitochondrial calcium and oxidative stress. The membrane potential is also increased compared to the other phases but lower than in healthy CEC. These cells resist the sustained need for more ATP caused by the depletion in surrounding cells in the tissue. The increase in mitochondrial mass leads to an increase in oxidative stress. At this stage, cells have reached their maximum capacity and start to cope with irreversible damage. (**3**) **Burnout stage**: These FECD CECs are showing a normal mitochondrial mass, and ΔΨm, ROS and mitochondrial calcium levels lower than CECs in the resistance stage. The loss in ΔΨm and mitochondrial calcium suggest the opening of the mitochondrial permeability transition pore, which leads to the initiation of apoptosis. (**4**) **Death stage**: Apoptotic FECD CECs with low mitochondrial mass, low oxidation stress and depleted ΔΨm. Mitochondrial damage leads to a loss in mitochondrial mass through mitophagy and loss of ΔΨm through the opening of the mitochondrial permeability transition pore. We believe that this better understanding of the chronology of events in FECD will make it possible to find new curative or preventive treatment for this pathology.

## Supplementary information

Supplementary materials.
