# Supplementary material for: Chronology of cellular events related to mitochondrial burnout leading to cell death in Fuchs endothelial corneal dystrophy
Source: Sci Rep. 2020 Apr 2;10:5811. doi: 10.1038/s41598-020-62602-x (PMC7118119; doi:10.1038/s41598-020-62602-x)
Supplement: Supplementary file 1 — Supplementary materials. [file 41598_2020_62602_MOESM1_ESM.pdf]

Supplementary material

Chronology of cellular events related to mitochondrial burnout leading to cell death in Fuchs endothelial corneal dystrophy

Sébastien Méthot[1,2], Stéphanie Proulx[1,2,3], Isabelle Brunette[4,5], Patrick J. Rochette[1,2,3,*]

1. Centre de Recherche du CHU de Québec – Université Laval, Axe Médecine Régénératrice, Hôpital du Saint-Sacrement, Québec, Canada
2. Centre de recherche en organogénèse expérimentale de l'Université Laval/LOEX, Québec, Canada
3. Université Laval, Faculté de Médecine, Département d'Ophtalmologie, Université Laval, Québec, Canada.
4. Maisonneuve-Rosemont Hospital Research Center, Montreal, Québec, Canada
5. Department of Ophthalmology, University of Montreal, Montreal, Québec, Canada

* Corresponding author:
Dr. Patrick J. Rochette
Centre de recherche du CHU de Québec – Université Laval
Axe médecine Régénératrice
Hôpital du Saint-Sacrement, Bureau H2-10
1050 Chemin Sainte-Foy, Québec, Qc, Canada, G1S 4L8
Phone: (418) 682-7568
E-mail: Patrick.rochette@orlo.ulaval.ca

Supplemental material and methods

UVB irradiation and apoptosis analysis

Corneal endothelial explants from cadaveric eyes were placed in PBS and irradiated or not with 40 kJ/m^2 UVB to induce apoptosis. UVB irradiation system consists of RPR-3000 UVB lamps (Southern New England Ultraviolet Co.) with an emission peak of 300 nm. A cellulose acetate sheet (Kodacel TA-407, clear 0.015 in.; Eastman-Kodak Co.) was used to filter out wavelength below 295 nm. Sixteen hours post-UVB, apoptosis was measured using CellEvent Caspase-3/7 Green detection assay (Invitrogen) at a concentration of 5 µM and mitochondria were labeled using Mitotracker Deep Red FM at a concentration of 80 nM (Invitrogen).

Supplemental figure

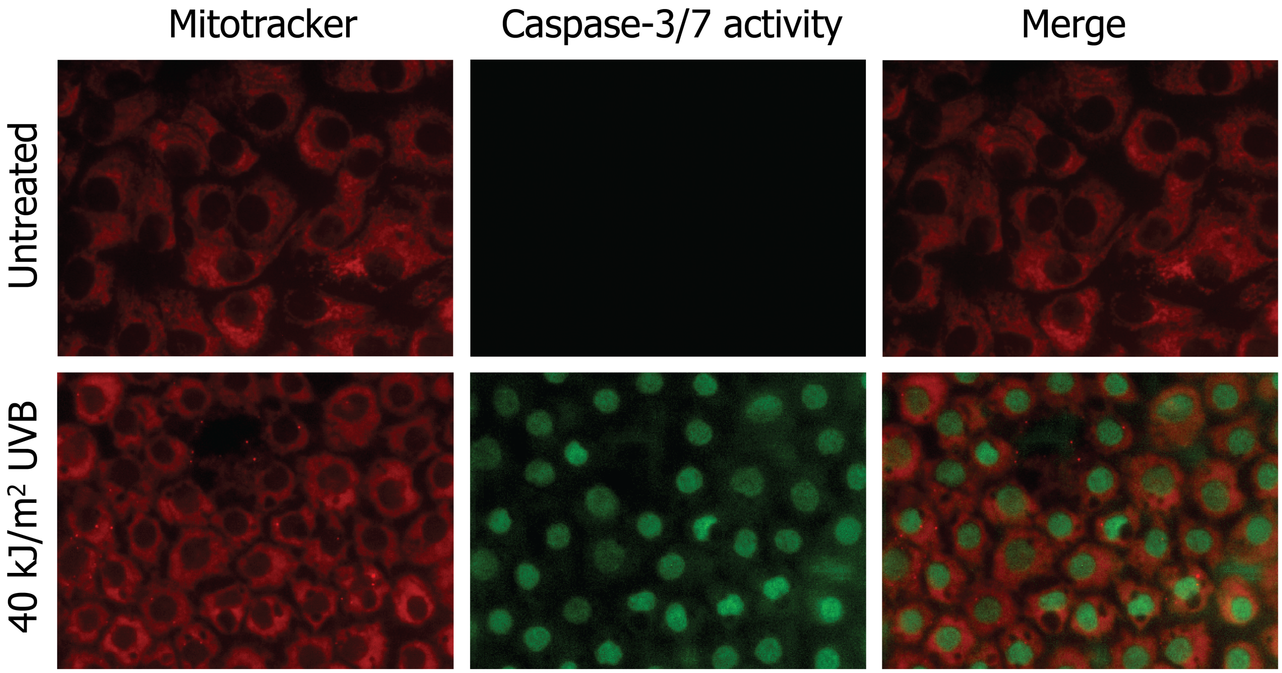

Fig S1: UVB-induced apoptosis in corneal endothelial explants. Corneal endothelial explants from cadaveric human eyes has been irradiated with 40 kJ/m2 UVB or unirradiated (untreated). Sixteen hours post-irradiation, a marker of cell death by apoptosis (caspase-3/7 activity; green) was used in conjunction with the mitochondrial mass marker (mitotracker; red). Nuclear green labeling of all cells in the explant following UVB-exposure shows a strong induction of apoptosis in corneal endothelial cells.

Supplemental table

Table S1. Classification of age and sex of healthy and FECD donors used in this study by figures.

Healthy (H) or FECD (F)	Age	Sex	Figure	Number of Fields
F	66	Male	2.6	8
F	73	Female	2.6	8
F	90	Female	2.6	11
H	70	Female	2.6	12
H	77	Female	2.6	8
H	78	Male	2.6	8
F	61	Female	3	8
F	71	Female	3	9
F	71	Female	3	8
H	64	Female	3	7
H	61	Female	3	11
H	82	Female	3	10
F	57	Female	4	7
F	72	Female	4	8
F	58	Male	4	10
F	67	Female	4	7
H	56	Male	4	7
H	60	Male	4	9
H	81	Female	4	11
F	55	Female	5	8
F	70	Male	5	9
F	74	Female	5	7
H	61	Female	5	7
H	64	Female	5	8
H	82	Female	5	11
H	66	Female	Sup 1	-
